# Effect of Microwave Radiation on the Properties of Hydrogel, Cork, Perlite, and Ceramsite

**DOI:** 10.3390/gels10080543

**Published:** 2024-08-22

**Authors:** David Průša, Stanislav Šťastník, Kateřina Svobodová, Karel Šuhajda, Zuzana Sochorová

**Affiliations:** 1 Faculty of Forestry and Wood Technology, Mendel University in Brno, Zemědělská 1665/1, 613 00 Brno, Czech Republic; zuzana.sochorova@mendelu.cz; 2 Institute of Building Structures, Faculty of Civil Engineering, Brno University of Technology, Veveří 331/95, 602 00 Brno, Czech Republic; stastnik.s@fce.vutbr.cz (S.Š.); 23215@vutbr.cz (K.S.); suhajda.k@fce.vutbr.cz (K.Š.)

**Keywords:** hydrogel, cork, ceramsite, perlite, microwave radiation

## Abstract

The present work analyzes the effect of releasing physically bound water from hydrogel, cork, perlite, and ceramsite on materials exposed to microwave radiation and subsequently investigates possible changes in the physical properties of these materials (water absorption and thermal conductivity coefficient). The release of physically bound water from individual materials has potential practical applications in materials engineering, for example, in the internal curing of concrete, where individual aggregates could, under the influence of microwave radiation, release water into the structure of the concrete and thus further cure it. Experimental analysis was carried out with samples of the above-mentioned materials, which were first weighed and then immersed in water for 24 h. Then, they were weighed again and exposed to microwave radiation. After exposure, the samples were weighed again, left immersed in water for 24 h, and weighed again. The focus of the study was on the ability of the aggregates to release water due to microwave radiation and on the changes in the properties (water absorption, thermal conductivity coefficient) of these materials when exposed to microwave radiation. The samples were further monitored by digital microscopy for possible changes in the surface layer of the materials. The hydrogels show the highest water absorption (1000%) and the fastest water release (45 min to complete desiccation). After the release of water due to microwave radiation, their ability to absorb water is maintained. Of interest, however, is that in the case of almost complete removal of water from the soaked hydrogel, the original powdered state of the hydrogel is not obtained, but the outcome has rather a solid structure. In the case of cork, the water absorption depends on the fraction of the material.

## 1. Introduction

The use of admixtures is the most effective way to change the quality of concrete and give it specific properties [1]. Certainly, admixtures in the strict sense of the word need not be used, but aggregates such as cork, perlite, polystyrene, or ceramsite can be utilized [2,3]. Furthermore, there is ongoing research where newer materials based on gel structure are becoming available, and hydrogel is one of such materials [4].

The effect of these materials on the physical and mechanical properties of concrete is well known, yet there is still room for further research, especially in terms of new technologies, such as accelerating the hardening process of concrete. One of these technologies is the use of microwave radiation in construction, which can accelerate the hardening and setting process of the binder component of concrete [5]. Concrete that has undergone accelerated curing will typically have lower strengths than a reference concrete that has not been subjected to acceleration [6,7].

One of the means of eliminating the change in the mechanical properties of concrete after accelerating its solidification can be the so-called internal curing of concrete [8], where aggregates soaked in water slowly release the water into the cement to ensure its hydration. Before using aggregates in concrete, it is however desirable to investigate the effect of microwave radiation on their properties and, in particular, how quickly and effectively they can release water into the concrete when exposed to microwave radiation and thus further hydrate it.

The main objective of this research is to analyze the effect of microwave radiation on the ability of hydrogels, cork, perlite, and ceramsite to release physically bound water and how this process affects their physical properties, such as water absorption and thermal conductivity coefficient. This information is key to understanding whether these materials can be effectively used for internal curing of concrete using microwave radiation and thus help limit the negative effect of this radiation on the compressive strength of concrete.

The results of this research may provide new insights into the use of microwave radiation in construction, particularly in the context of accelerated concrete curing. If it can be proved that selected aggregates can effectively release water when exposed to microwave radiation and thus contribute to better hydration of cement paste, this could lead to the development of new technologies and processes for the production of high-strength concrete that is less susceptible to the negative effects of accelerated setting. This would improve the efficiency and quality of concrete structures, which would have a wide impact on the construction industry.

Internal curing of concrete is a specific enhancement of chemical reactions in hydrated cement paste and concrete. This process ensures the release of water during the hydration of the cement paste by providing sufficient water over time. This prevents water loss during hydration (e.g., when the cement paste is exposed to high temperatures) and ensures that water is released evenly throughout the curing process [9,10,11,12].

Internal curing was originally defined by the American Concrete Institute (hereinafter referred to as ACI) as “supplying water throughout a freshly placed cementitious mixture using reservoirs, via prewetted lightweight aggregates, that readily release water as needed for hydration or to replace moisture lost through evaporation or self-desiccation” [13].

In 2013, ACI changed the definition of internal curing to the “process by which the hydration of cement continues because of the availability of internal water that is not part of the mixing water” [14].

Internal curing was first used in 1918 by the U.S. government [15] in the construction of concrete ships and is now widely used throughout Europe, Japan, Russia, the United States, and other parts of North and South America. Internal curing is mainly used in lightweight concrete, though it is also used in many other applications such as green roofs, horticulture, asphalt chippings, lightweight geotechnical fill, etc. [16].

Internal curing helps the concrete reach its maximum potential in a simple, economical, and sustainable way. It improves hydration, reduces chloride penetration, microcracking, and twisting, and increases durability.

While designing concrete specifically for internal curing is relatively new, the concept of using lightweight aggregates for improving the hydration of cement paste was observed as early as the 1950s by Paul Klieger [17,18].

Research into the deliberate use of lightweight aggregates for internal curing of concrete began to take shape in the late 1990s when several research groups, mainly in Europe [19,20,21], began to actively explore whether it is possible to design mixtures for internal curing using prewetted lightweight aggregates.

This was followed by the development of design procedures that enabled the calculation of both the spatial distribution and the quantity of prewetted lightweight aggregates [22]. Internal curing has thus become a developed technology, and its use has started to grow as it provides new possibilities for concrete structures.

Concrete, as the most widely used building material known to mankind [23], still in many cases falls short of its true potential. It is possible to minimize this by using a relatively old technology of internal curing, which dates back to the times of the Roman Empire when concrete was built using volcanic materials and lightweight aggregates [23,24,25]. Based on research conducted by several authors [26,27,28], we are now able to understand better how internal curing works and how to engineer it properly.

Internal curing of concrete provides something that is needed in most cases and that conventional treatments cannot provide: additional internal water, which helps to prevent early shrinkage and increases the hydration of cementitious materials throughout the entire material. Once the concrete has set, hydration partially fills the pores in the cementitious material, which otherwise causes stresses leading to shrinkage. This reduces shrinkage, cracking, and early twisting/deformation, increases strength, and reduces the permeability of the concrete, making it more resistant to chloride penetration [29,30,31,32,33,34].

Internal curing has proven to work well with supplementary cementitious materials especially at higher doses, as fly ash, slag, and silica fume have an increased water requirement in their reaction compared to hydrating Portland cement [31,32].

Internal curing does not replace conventional surface treatments but rather acts in conjunction with them to make the concrete more durable. It can also help to offset less-than-ideal weather conditions and the inadequate conventional treatments that are often found in the real world.

Internal curing is being promoted in all areas of concrete construction, including concrete pavement, concrete flatwork, bridges, structural elements, pavers, and mass concrete applications [29,30,31].

The various materials that are incorporated into buildings should have the ability to be reused as part of recycling. As building materials are usually assembled into functional material composites and may also be coated with different surface treatments, it is important to separate these composites from each other using appropriate technologies when recycling. Conventional treatment by crushing the materials in a crusher usually does not completely succeed in separating them from each other. This offers the scope for the use of methods that can separate composites at their material interface. Such separation of the composites must not compromise their material properties. Therefore, the individual materials most commonly used in the construction industry are studied in terms of their microwave radiation loading. The effect of microwave radiation on specific building materials is examined from a broader perspective; the research aims to clarify the effect of microwave radiation on critical parameters of building materials.

Microwave radiation (EMW) is one of the most favored tools for heating dielectric materials not only in the industrial sector but also in the everyday life of a general household. One of its possible applications is to accelerate the solidification of silicate binders, for example, in the case of cement. The advantages provided by microwave radiation lead to selective bulk heating—only polar substances (e.g., water) are able to absorb microwave radiation. Due to this phenomenon, the rate of hardening of the concrete can increase, and thus the handling strength is obtained a lot faster. The technology is mainly used in the production of precast concrete panels. However, in recent years, research on accelerating the solidification of concrete by microwave radiation has only been marginally developed. The main reason lies in the generally lower strength values of concrete that has been subjected to accelerated solidification [35,36,37,38].

Microwaves refer to the section of electromagnetic radiation with frequencies ranging from 300 MHz to 300 GHz, which corresponds to wavelengths from 1 mm to 1 m. Other frequencies are also allowed for industrial purposes, but in the field of civil engineering, the frequency of 2450 MHz with a wavelength of 12.2 cm is of particular interest. Microwave radiation causes heating of the material, whereby the heated molecules are oriented according to their polarity in the electric field. When microwave radiation is applied to water molecules, electromagnetic energy is transformed, and the molecules are heated. Subsequently, the other components of the building material are heated as well. The use of microwave equipment in a proper way is very safe, and harmful effects on humans can only occur if the equipment is not handled correctly or if there is direct exposure at very close range for at least several minutes. Strong microwave radiation poses a higher risk to human health and is hence restricted by several regulations, including those of the European Union (Directive 2004/40/EC). Some countries have also introduced their own restrictions; these typically set a permissible electromagnetic field strength (from 7 V·m to 61 V·m) or power density (from 0.1 W·m^−2^ to 10 W·m^−2^) [39,40,41].

## 2. Results and Discussion

From the individual experiments, it can be seen that hydrogels show the highest value of water absorption (both types of hydrogels about 1000%), but at the same time, when exposed to microwave radiation, water is released from them the fastest. It took 450 min for the full release of water from the samples.

In the case of cork, the value of water absorption varied depending on the aggregate fraction, but even though sample 1, fine cork, has an absorption of 844%, the release of water bound in the material is slower than in the case of hydrogels. It took 540 min for the full release of water from the samples.

The crushed ceramsite has a water absorption of 91.5%, and the water was released from the sample gradually over approximately the same time as from the hydrogel samples.

The perlite has a water absorption of 179%, and the water was similarly released from the sample as from the hydrogel samples.

**Figure 1:**
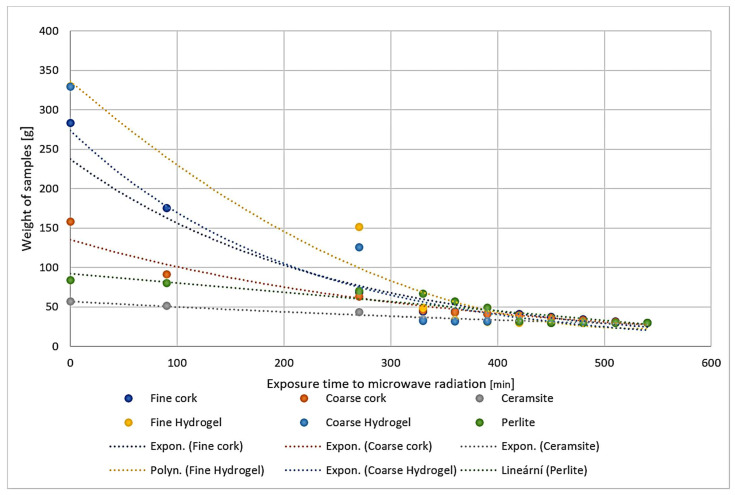
Representation of the drying process of samples during exposure to microwave radiation.

The entire drying process of the individual material samples can be observed in the summary of Figure 1. Here, we observe the individual curves that symbolize the masses of the individual materials in the specific drying times. The hydrogels release water the fastest, while the fine cork and ceramsite release water slowly. Individual samples were weighed at 90 min, 270 min, 330 min, 360 min, 390 min, 420 min, 450 min, 480 min, 510 min, and 540 min of the microwave exposure.

Before exposure to microwave radiation, the hydrogels, both fine and coarse, were able to bind water immediately; however, after exposure to microwave radiation, even though this ability to bind was retained, they bound water more slowly. Thus, during the hydration of concrete, it is possible that the hydrogel may release water during exposure to microwave radiation but bind it again at later stages of hydration, and with this, we can expect some volume changes that could under certain conditions disturb the cement matrix. In general, therefore, it can be assumed that the hydrogel has reversible properties to a certain extent.

**Figure 2:**
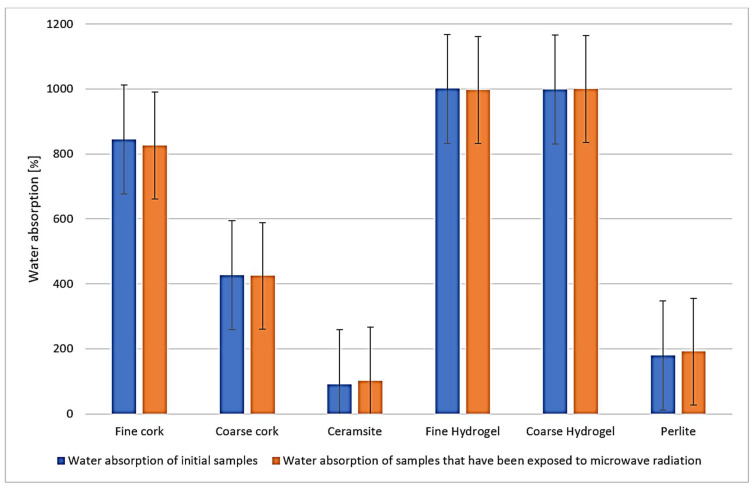
Representation of absorbance values of samples before and after exposure to microwave radiation.

**Figure 3:**
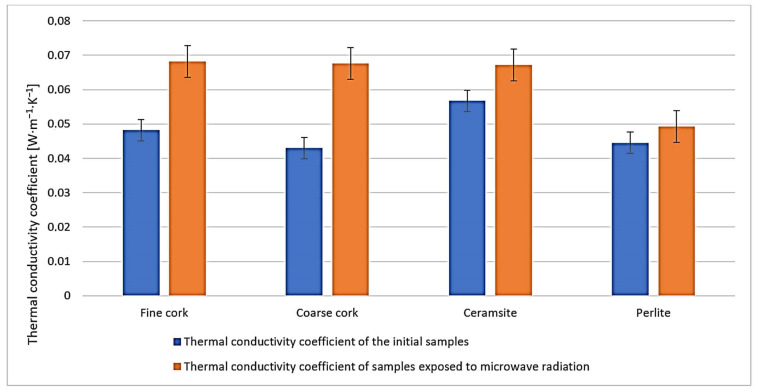
Representation of thermal conductivity coefficient values before and after exposure to microwave radiation.

We do not observe significant changes in the water absorption characteristics of the samples before and after exposure to microwave radiation (Figure 2). The most significant changes can be observed in the values of the thermal conductivity coefficient (Figure 3), where for all samples, the value of the thermal conductivity coefficient is higher after exposure to microwave radiation. However, from the measured data, it is currently difficult to determine whether it is due to a change in the microstructure of the samples caused by exposure to microwave radiation or whether it is due to residual water in the samples.

**Figure 4:**
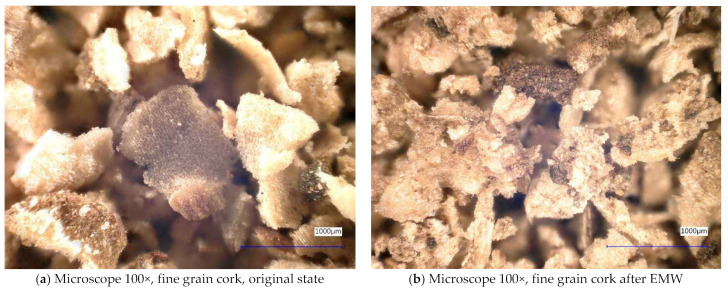
Digital microscope images of the fine cork sample during the experiment (Zoom 100×).

**Figure 5:**
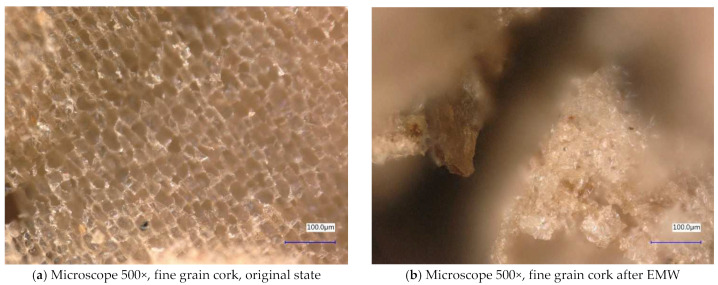
Digital microscope images of the fine cork sample during the experiment (Zoom 500×).

**Figure 6:**
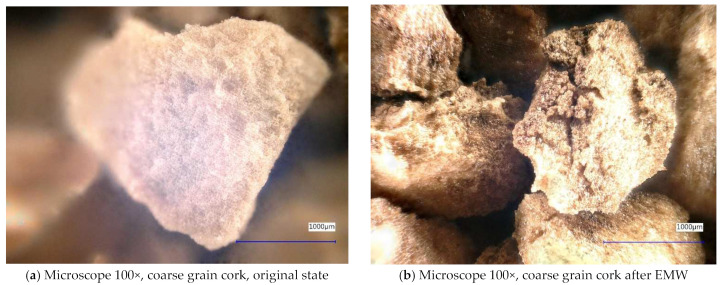
Digital microscope images of the coarse cork sample during the experiment (Zoom 100×).

**Figure 7:**
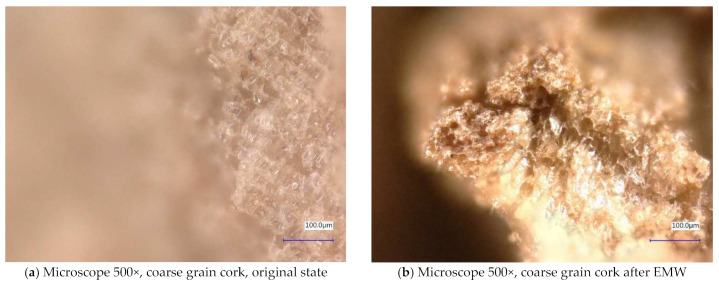
Digital microscope images of the coarse cork sample during the experiment (Zoom 500×).

**Figure 8:**
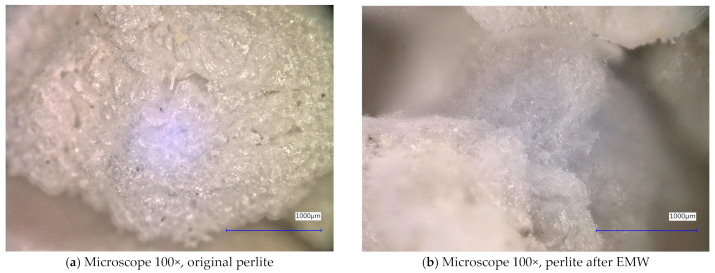
Digital microscope images of the perlite sample during the experiment (Zoom 100×).

**Figure 9:**
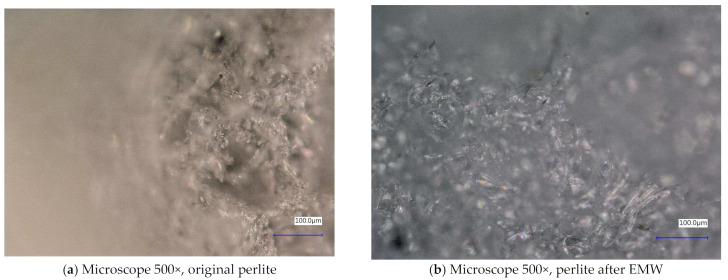
Digital microscope images of the perlite sample during the experiment (Zoom 500×).

**Figure 10:**
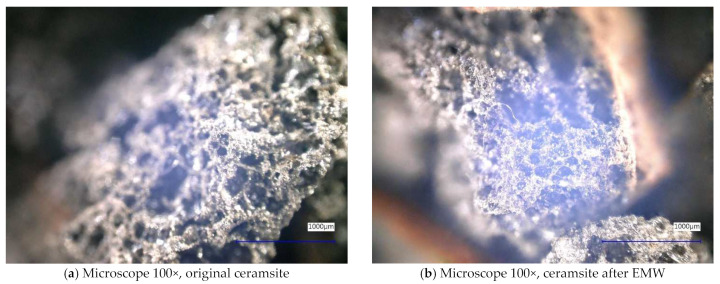
Digital microscope images of the ceramsite sample during the experiment (Zoom 100×).

**Figure 11:**
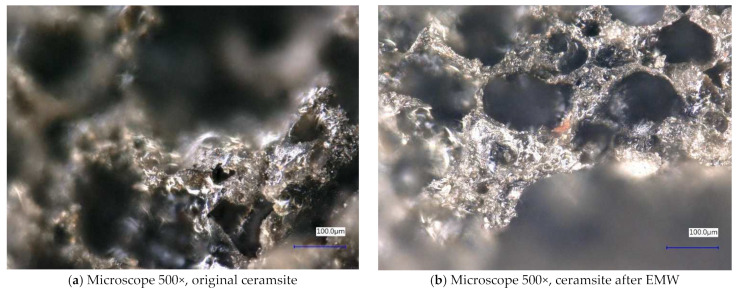
Digital microscope images of the ceramsite sample during the experiment (Zoom 500×).

**Figure 12:**
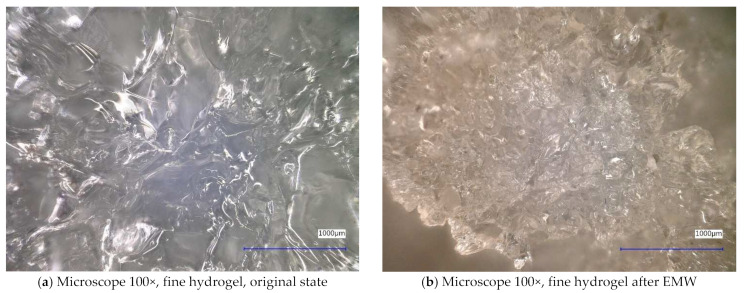
Digital microscope images of the fine hydrogel sample during the experiment (Zoom 100×).

**Figure 13:**
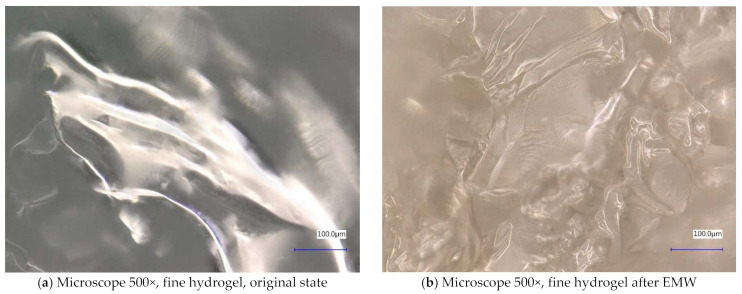
Digital microscope images of the fine hydrogel sample during the experiment (Zoom 500×).

**Figure 14:**
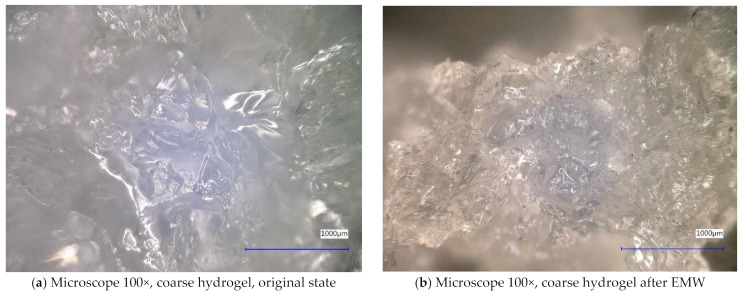
Digital microscope images of the coarse hydrogel sample during the experiment (Zoom 100×).

**Figure 15:**
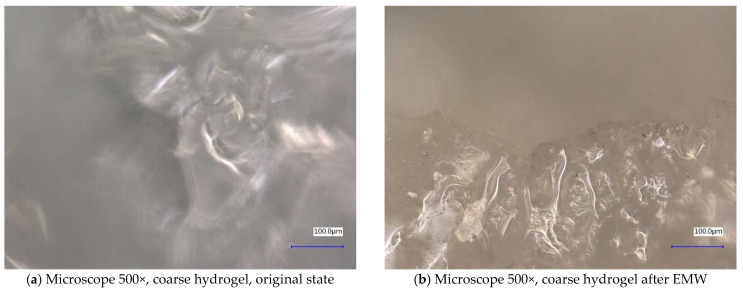
Digital microscope images of the coarse hydrogel sample during the experiment (Zoom 500×).

**Figure 16:**
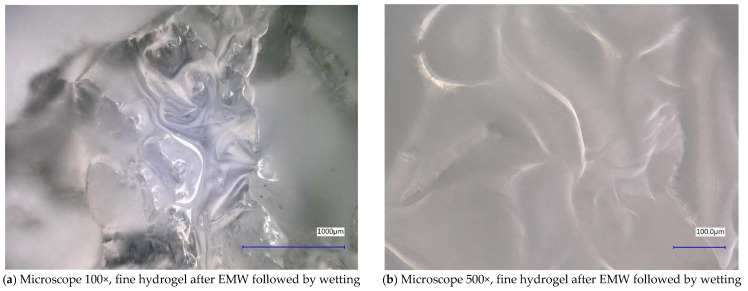
Digital microscope images of the fine hydrogel sample after EMW followed by wetting.

**Figure 17:**
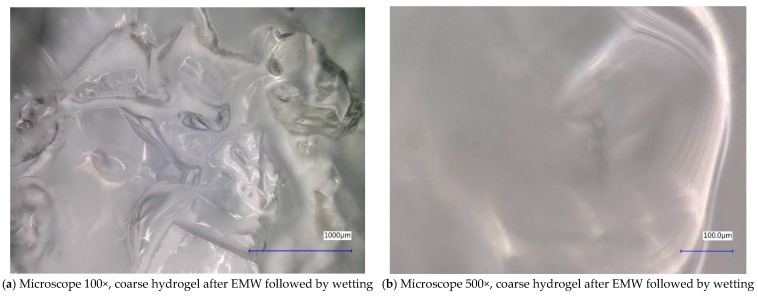
Digital microscope images of the coarse hydrogel sample after EMW followed by wetting.

Next, the individual samples were observed using a digital microscope at 100× and 500× magnification (Figure 4, Figure 5, Figure 6, Figure 7, Figure 8, Figure 9, Figure 10, Figure 11, Figure 12, Figure 13, Figure 14, Figure 15, Figure 16 and Figure 17). In Figure 4a, we see an image taken through a digital microscope at 100× magnification of the original fine cork before exposure to microwave radiation, and in Figure 4b, the same sample of fine cork but after exposure to microwave radiation. Similarly, in Figure 5a, we observe the same image of fine cork before microwave exposure but at 500× magnification. Figure 5b shows the same type of cork taken with a digital microscope at 500× magnification but after exposure to microwave radiation. From the images taken, it can be concluded that exposure to microwave radiation has no observable effect on the structure of fine cork under the experimental conditions.

Figure 6 and Figure 7 show samples of coarse cork taken with a digital microscope. These images are of the coarse cork before microwave exposure (Figure 6a and Figure 7a) taken at magnifications of 100× and 500×, respectively, and images taken after microwave exposure (Figure 6b and Figure 7b) at the same magnifications.

In the digital microscope images, we do not detect significant changes in the surface microstructure of the samples, except in Figure 6b, where we can observe a crack in the granule of the cork. However, it is not clear whether this is a crack caused by exposure to microwave radiation or a crack from the production process. No similar cracks have been observed on other cork granules.

In Figure 8 and Figure 9, we observe perlite samples taken with a digital microscope before and after exposure to microwave radiation. From these images, it can be concluded that microwave radiation does not affect the structure of the samples.

In Figure 10 and Figure 11, we observe samples of ceramsite taken by digital microscope before and after exposure to microwave radiation. The ceramsite samples that were exposed to microwave radiation show a different pore size compared to the samples that were not exposed to microwave radiation. The different pore sizes in the samples may be due to the diffusion of water vapor during the experiment, which may have caused the increase in pore size.

**Figure 18:**
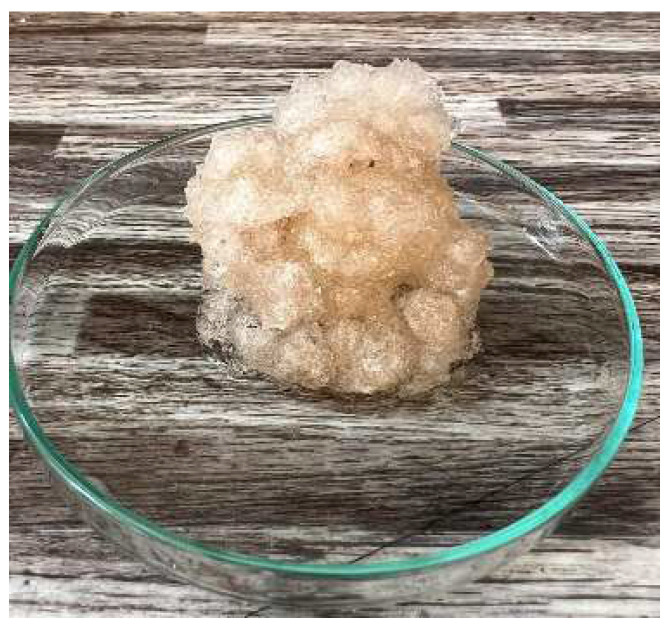
Photograph of the coarse hydrogel sample exposed to microwave radiation and for 5 min placed again in a water bath.

**Figure 19:**
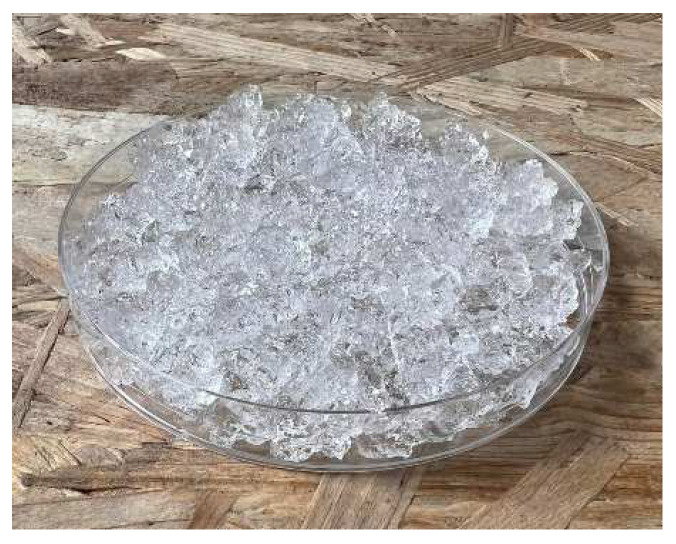
Photograph of the coarse hydrogel sample exposed to microwave radiation and for 480 min again placed in a water bath.

**Figure 20:**
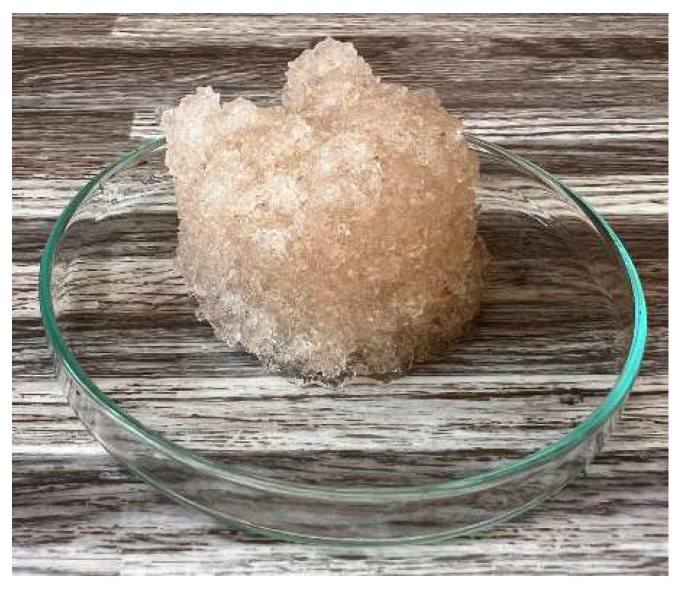
Photograph of the fine hydrogel sample exposed to microwave radiation and for 5 min placed again in a water bath.

**Figure 21:**
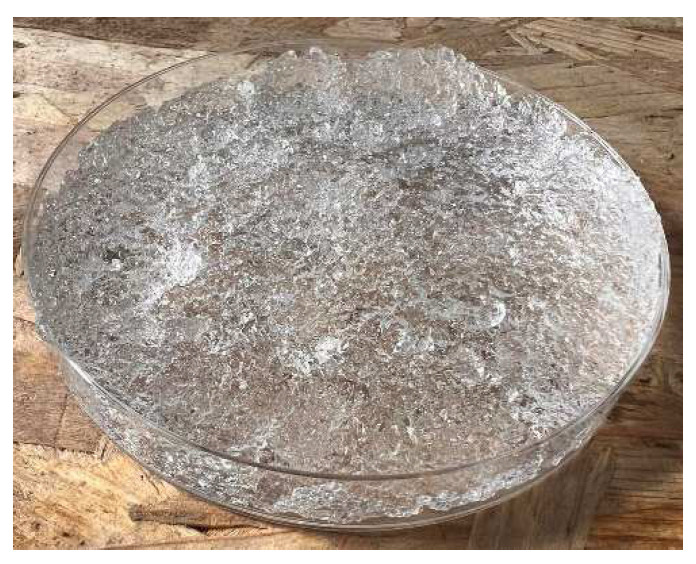
Photograph of the fine hydrogel sample exposed to microwave radiation and for 480 min again placed in a water bath.

In Figure 12 and Figure 13, we observe samples of fine hydrogel (12–15) and coarse hydrogel (14–15) taken with a digital microscope at magnifications of 100× and 500× before and after exposure to microwave radiation. The images show the shrinkage of the individual hydrogels caused by microwave exposure. However, in the case of hydrogel, this is a reversible property, i.e., the hydrogel regains its gel structure when water is added (see Figure 18, Figure 19, Figure 20 and Figure 21).

In Figure 12, Figure 13, Figure 14, Figure 15, Figure 16 and Figure 17, we observe digital microscope images of hydrogel samples at 100× and 500× magnification. These are samples of hydrogel that was mixed with water, obtained a gel-like structure, was dried, and then water was again added to the dried hydrogel. This phenomenon will be further investigated experimentally. In Figure 18, Figure 19, Figure 20 and Figure 21, we observe pictures of these hydrogel samples after exposure to microwave radiation (Figure 18 and Figure 20) and after the re-addition of water (Figure 19 and Figure 21). The hydrogels that were exposed to microwave radiation show a longer time required for water absorption compared to the hydrogels before microwave exposure.

### 2.1. The Course of the Experiment

The aggregates used in the experiment were ceramsite, two types of cork (according to the fraction), perlite, and two types of hydrogels (again, according to the fraction). The experiments seek to verify the effect of microwave radiation on the different materials. 

**Figure 22:**
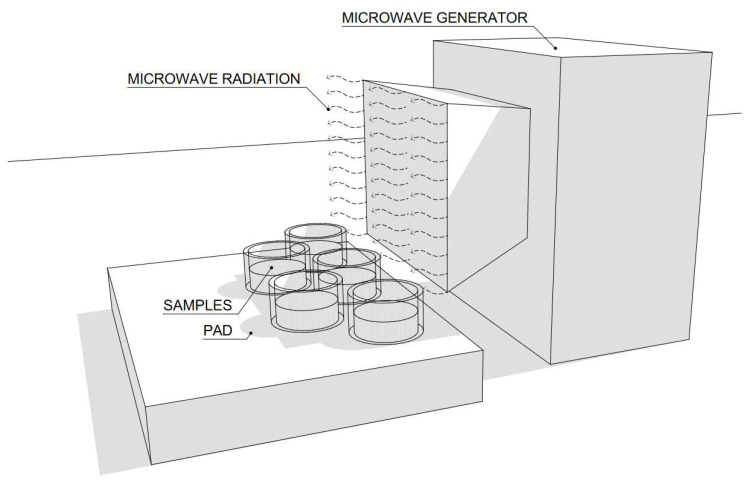
Depiction of the process of exposing the samples to microwave radiation.

First, a 30 g portion of each sample was placed in laboratory dishes. Subsequently, the samples were fully immersed in 300 g of water for 24 h. After this time, the remaining water was poured off and the water-soaked samples were weighed. The water absorption of the samples was calculated. The soaked samples were exposed to microwave radiation at 750 W for 540 min, as shown in Figure 1, and the moisture loss was continuously measured by weighing. The drying progress can be observed in Figure 1 and the experiment progress is displayed in Figure 22.

After the samples were dried to a constant weight, they were subjected to surface microstructure analysis under a microscope, immersed in water for 24 h, and then weighed. The water absorption after microwave radiation was calculated.

**Figure 23:**
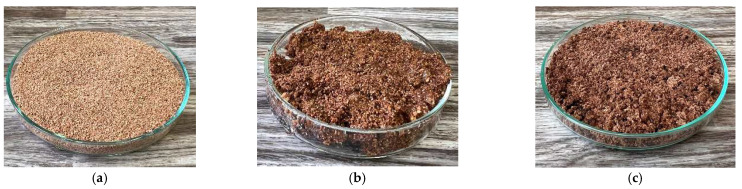
Photographs of the fine cork sample during the experiment. (**a**) Photograph of cork sample 1 before wetting. (**b**) Photograph of cork sample 1 after wetting. (**c**) Photograph of cork sample 1 after exposure to microwave radiation.

**Figure 24:**
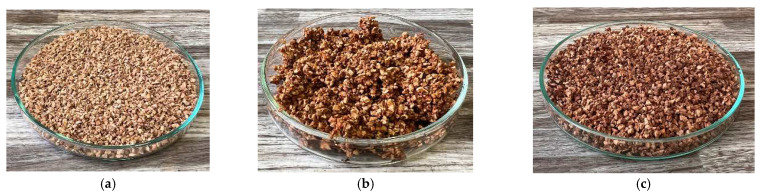
Photographs of the coarse cork sample during the experiment. (**a**) Photograph of cork sample 2 before wetting. (**b**) Photograph of cork sample 2 after wetting. (**c**) Photograph of cork sample 2 after exposure to microwave radiation.

**Figure 25:**
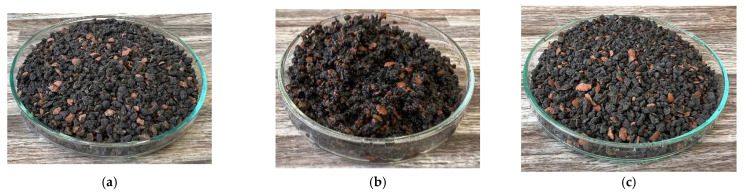
Photographs of the ceramsite sample during the experiment. (**a**) Photograph of ceramsite sample before wetting. (**b**) Photograph of ceramsite sample after wetting. (**c**) Photograph of ceramsite sample after exposure to microwave radiation.

**Figure 26:**
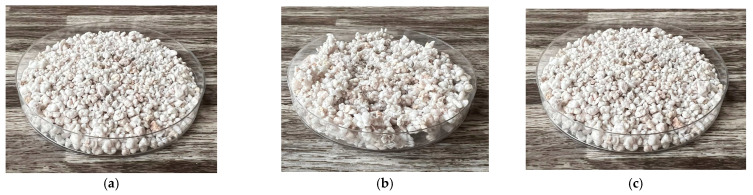
Photographs of the perlite sample during the experiment. (**a**) Photograph of perlite sample before wetting. (**b**) Photograph of perlite sample after wetting. (**c**) Photograph of perlite sample after exposure to microwave radiation.

**Figure 27:**
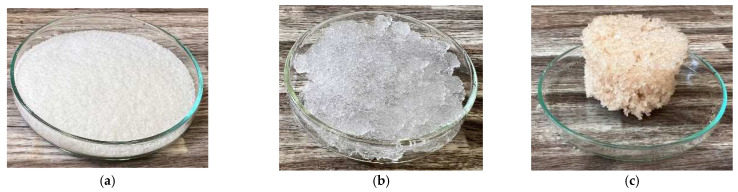
Photographs of the fine hydrogel sample during the experiment. (**a**) Photograph of fine hydrogel sample before wetting. (**b**) Photograph of fine hydrogel sample 1 min after wetting. (**c**) Photograph of fine hydrogel sample after exposure to microwave radiation.

**Figure 28:**
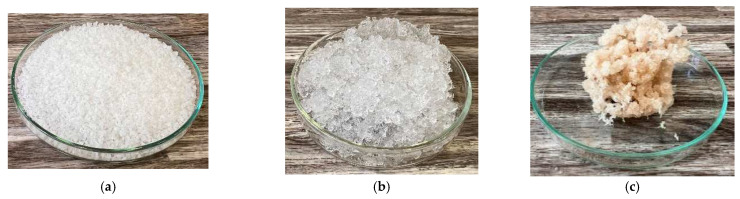
Photographs of the coarse hydrogel sample during the experiment. (**a**) Photograph of coarse hydrogel sample before wetting. (**b**) Photograph of coarse hydrogel sample 1 min after wetting. (**c**) Photograph of coarse hydrogel sample after exposure to microwave radiation.

In addition, the thermal conductivity coefficient of the individual cork, ceramsite, and perlite samples was measured with a non-stationary method before and after exposure to microwave radiation. Figure 23, Figure 24, Figure 25, Figure 26, Figure 27 and Figure 28 show the individual materials before wetting, after wetting, and after exposure to microwave radiation.

### 2.2. Placement in a Water Bath

The samples were first weighed and then placed in a drying chamber at 105 °C (except for the hydrogel) for 24 h and then weighed again. They were then placed in a water bath, which was placed in a tempered chamber once again for 24 h. The water was maintained at a constant temperature of 20 ± 2 °C. The test samples were then removed from the bath and subjected to experimental tests.

### 2.3. Determination of Bulk Density (ČSN ISO 60 (642101))

The bulk density of the bulk materials was determined by measuring the volume of the test sample in the container and then determining its weight. The preparation of the test samples, the test procedure, and the evaluation were carried out following the relevant standard ISO EN ISO 60 (642101) Plastics—Determination of apparent density of material that can be poured from a specified funnel [42].

### 2.4. Determination of Water Absorption (ČSN EN ISO 16535 (727056))

The experimental samples were weighed and then placed in an oven at 105 °C for 24 h. After the required time elapsed, the test specimens were retrieved and, after cooling to room temperature, weighed again. The samples were placed in pans that were later filled with water and sealed with a lid. The containers were filled 2 cm below their rim. After 24 h, the samples were retrieved and weighed again [43].

### 2.5. Determination of the Thermal Conductivity Coefficient (ČSN 72 7306)

The test samples were stored in a drying chamber at 105 °C for 24 h. After the required time elapsed, the samples were retrieved, and after cooling to room temperature, they were placed in molds, and the determination of the thermal conductivity coefficient was carried out using the ISOMET 2104 non-stationary method [44].

## 3. Conclusions

Based on the experiments performed and their evaluation, it was determined that hydrogel, cork, perlite, and ceramsite release physically bound water when exposed to microwave radiation. The measurements show that exposure of the samples to microwave radiation ensures the release of physically bound water within a short time (maximum 540 min) as water vapor, with no significant changes in the samples’ properties. Hydrogel showed a change in water-binding capacity, reflected in a prolonged water-binding time after microwave exposure.

Future research will focus on exposing hydrogel to modified microwave radiation and its further behavior, potentially extending to its use for internal curing of concrete. It is possible that, in the case of unsuitable methodology, the aggregates could release water into the concrete and help to hydrate it in the short term, but then subsequently take up the water needed for hydration, having the opposite effect.

In addition to the ability to release water, changes in water absorption and thermal conductivity coefficient were investigated. The results show that the water absorption values after microwave radiation are comparable to the original values. The values of the thermal conductivity coefficient increase, but this may be due to residual moisture in the samples, not due to microwave radiation.

Digital microscope images show no significant changes in the surface microstructure of the materials. This is important since the microwave radiation does not compromise the properties of the used fillers.

Microwave radiation can cause the release of water from water-soaked aggregates in concrete, potentially aiding hydration and reducing the negative effects of accelerated curing. However, further research is needed to support these hypotheses.

Moreover, to comprehensively evaluate the effect of adding water-soaked aggregates to the concrete mix, it is necessary to also study the resulting material in terms of the environmental impact of its life cycle, taking into account the balance of overall water management.

It is noteworthy that we have not found similar research in the literature that focuses on a comparative analysis of these materials using internal curing of concrete and microwave radiation. Most studies to date have focused on the direct application of these materials to concrete mixes without analyzing in detail their individual properties and effectiveness when exposed to microwave radiation. Hydrogels and cork have the highest water-holding capacity; thus, future research should verify if they can improve the compressive strength and other mechanical properties of concrete when microwave radiation is used to accelerate curing.

## 4. Materials and Methods

**Figure 29:**
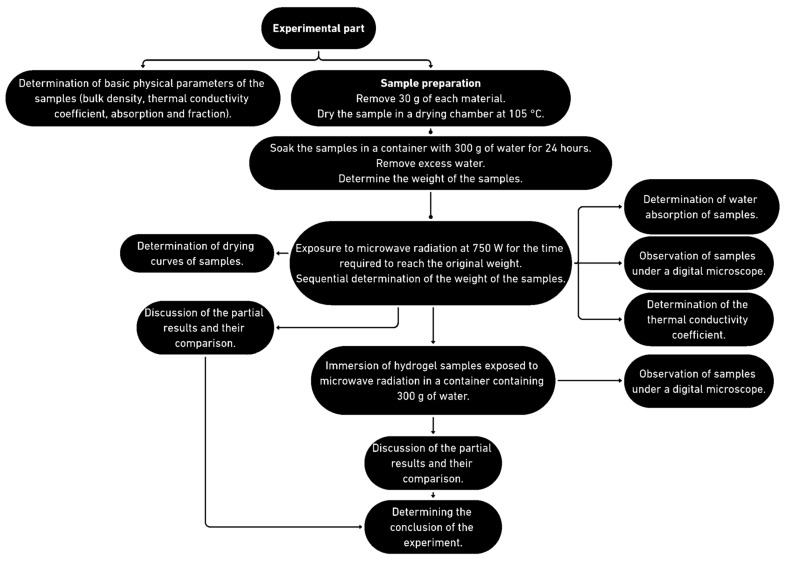
The display of the algorithm of the experiment.

For the purpose of the experiment, cork, ceramsite, and perlite were selected as aggregates. In addition to these materials commonly available and widely used in the construction industry, hydrogel was also examined. The experiment aims to verify the possibility of releasing physically bound water from individual samples utilizing microwave radiation. The course of the experiment is described in detail in the section below, the properties of the individual materials can be observed in Table 1, and the progress of the experimental part can be observed in Figure 29.

### 4.1. Cork

The cork industry uses more than 280,000 tons of cork every year, about a quarter of which is used in the construction industry. However, approximately 20–30% of the raw cork entering processing plants ends up as discarded material, mainly in the form of cork dust. Finding useful applications for this discarded cork can have a significant economic and environmental impact. Due to its diverse mechanical and physical properties, cork has become a versatile material with a wide range of potential applications [45].

Two samples of cork were used for the experiment. Sample 1 is cork with a fraction of 0.5–1 mm, and sample 2 is cork with a fraction of 1–2 mm.

### 4.2. Ceramsite

Lightweight expanded clay aggregate (LECA) or ceramsite is a lightweight aggregate produced in a rotary kiln by heating clay to a temperature of approximately 1200 °C. The heating process causes gases trapped in the clay to expand, creating thousands of little bubbles and thus giving the material a porous texture. Due to the circular motion in the kiln, LECA is roughly round or oblong and is available in a variety of sizes and densities. Besides other purposes, LECA is used to make lightweight concrete products.

For the needs of the experiment, crushed ceramsite with a fraction of 0.5–1 mm was used.

### 4.3. Hydrogel

Hydrogels are three-dimensional (3D) structures of relatively uncomplicated design. They are composed mainly of long polymer chains that form a complex matrix in which the spaces between the polymer chains are filled with water molecules. Depending on the chemical nature of the polymer and the degree of its crosslinking, the properties of hydrogel matrices considerably vary. Hydrogels are widely used in medicine and pharmaceutical sciences. They are used in regenerative medicine and tissue engineering and, in particular, as drug carriers [46], in wound dressings [47,48], or contact lenses [49,50].

However, hydrogels have applications in other industries as well, for example, hydrogels have been found to reduce autogenous shrinkage of high-performance concrete, and although some researchers have noted a decrease in strength at an early age after the addition of hydrogels, the strength was restored to control levels after sufficient time [51,52].

### 4.4. Perlite

Perlite is an amorphous material consisting mainly of silica [53]. Expanded perlite is used on its own as a granular thermal insulation material for horizontal insulation, i.e., mainly in ceilings and attics. It is also used as a component of mortars, plasters, and coatings with insulating properties and is used under normal temperature conditions [54,55]. Its advantageous properties are low bulk density (30–190 kg∙m^−3^) and low value of thermal conductivity coefficient, i.e., in the range of 0.04–0.05 W∙m^−1^∙K^−1^ [56].

### 4.5. Microwave Radiation Source

In the experiment, a portable microwave generator, Romill G1/2011, was used to irradiate the samples. This generator operates at a voltage of 230 V and a frequency of 50 Hz. The input power of the device is 1.5 kW, while the maximum microwave radiation power reaches 1 kW with a microwave frequency of 2450 MHz. For the purpose of this experiment, the device has been specifically set to output 750 W. Cyclic drying using this generator eliminates air pocket formation and ensures uniform heating of the material in depth, which is crucial for efficient drying of the samples [40,41].

## Figures and Tables

**Table 1:** Physical properties of used materials.

Sample Identification	Bulk Density [kg·m^−3^]	Fraction [mm]	Absorption [%]
Sample 1: fine cork	75 ± 5	0.5–1	800 ± 50
Sample 2: coarse cork	80 ± 5	1–2	400 ± 30
Ceramsite	500 ± 10	1–2	90 ± 10
Sample 1: fine hydrogel	100 ± 5	0.5–1	1000 ± 70
Sample 2: coarse hydrogel	100 ± 5	1–2	1000 ± 70
Perlite	60 ± 5	0.5–1	180 ± 20

## Data Availability

All data and materials are available on request from the corresponding author. The data are not publicly available due to ongoing research using a part of the data.

## References

[1] Akchurin T., Tukhareli V., Pushkarskaya O. (2016). The Modifying Additive for Concrete Compositions Based on the Oil Refinery Waste. Procedia Eng..

[2] Branco F.G., Tadeu A. (2007). Can cork be used as a concrete aggregate?. Int. J. Hous. Sci. Its Appl..

[3] Vijayalakshmi R., Ramanagopal S. (2018). Structural concrete using expanded clay aggregate: A review. Indian J. Sci. Technol..

[4] Krafcik M.J., Macke N.D., Erk K.A. (2017). Improved Concrete Materials with Hydrogel-Based Internal Curing Agents. Gels.

[5] Průša D., Šťastník S., Šuhajda K., Svobodová K., Žajdlík T., Hobzová K., Novotný M. (2023). The Effect of Microwave Radiation on the Solidification of C-S-H Gels: Its Influence on the Solidified Cement Mixtures. Gels.

[6] Yang K.-H., Mun J.-S., Cho M.-S. (2015). Effect of Curing Temperature Histories on the Compressive Strength Development of High-Strength Concrete. Adv. Mater. Sci. Eng..

[7] Yousuf S., Shafigh P., Ibrahim Z., Hashim H., Panjehpour M. (2019). Crossover Effect in Cement-Based Materials: A Review. Appl. Sci..

[8] Al Saffar D.M., Al Saad A.J., Tayeh B.A. (2019). Effect of internal curing on behavior of high performance concrete: An overview. Case Stud. Constr. Mater..

[9] ACI Commettee (2001). 308R-Guide to Curing Concrete.

[10] Bentz D.P., Weiss W.J. (2011). Internal Curing: A 2010 State-of-the-Art Review.

[11] Bentz D.P., Lura P., Roberts J.W. (2005). Mixture proportioning for internal curing. Concr. Int..

[12] Bentz D.P. (2007). Internal curing of high-performance blended cement mortars. Mater. J..

[13] Bentz D. (2013). Internal Curing: Curing Concrete from the Inside Out. Conference: ACI webinar. https://www.researchgate.net/publication/320564265_Internal_Curing_Curing_Concrete_from_the_Inside_Out/citations.

[14] ACI Commettee (2014). 213-03R Guide for Structural Lightweight-Aggregate Concrete.

[15] Panwar S., Jindal A. (2023). A review on self-curing agents. Innov. Infrastruct. Solut..

[16] Vosoughi P., Taylor P., Ceylan H. (2017). Impacts of Internal Curing on the Performance of Concrete Materials in the Laboratory and the Field.

[17] Rahman S. (2016). Investigation of Concrete Properties with Brick Chips as Internal Curing Medium. Master’s Thesis.

[18] Henkensiefken R. (2008). Internal Curing in Cementitious Systems Made with Saturated Lightweight Aggregate. Ph.D. Dissertation.

[19] Weiss J., Bentz D., Schindler A., Lura P. (2012). Internal curing. Structure.

[20] Kovler K., Jensen O.M. Activities of RILEM technical committee: Internal curing of concrete and anticipated research. Proceedings of the ACI Fall 2007 Convention.

[21] Sharma R., Jang J.G., Hu J.W. (2022). Phase-change materials in concrete: Opportunities and challenges for sustainable construction and building materials. Materials.

[22] Bentz D.P., Stutzman P.E. (2008). Internal curing and microstructure of high-performance mortars. ACI SP-256, Internal Curing of High Performance Concretes: Laboratory and Field Experiences.

[23] Bogas J.A., Gomes T. (2015). Mechanical and durability behaviour of structural lightweight concrete produced with volcanic scoria. Arab. J. Sci. Eng..

[24] Boarder R.F., Owens P.L., Khatib J.M. (2016). The sustainability of lightweight aggregates manufactured from clay wastes for reducing the carbon footprint of structural and foundation concrete. Sustainability of Construction Materials.

[25] Thienel K.C., Haller T., Beuntner N. (2020). Lightweight concrete—From basics to innovations. Materials.

[26] Yang L., Shi C., Liu J., Wu Z. (2021). Factors affecting the effectiveness of internal curing: A review. Constr. Build. Mater..

[27] Esteves L.P. (2009). Internal Curing in Cement-Based Materials. Ph.D. Thesis.

[28] Vázquez-Rodríguez F.J., Elizondo-Villareal N., Verástegui L.H., Arato Tovar A.M., López-Perales J.F., Contreras de León J.E., Gómez-Rodríguez C., Fernández-González D., Verdeja L.F., García-Quiñonez L.V. (2020). Effect of mineral aggregates and chemical admixtures as internal curing agents on the mechanical properties and durability of high-performance concrete. Materials.

[29] Kioumarsi M., Azarhomayun F., Haji M., Shekarchi M. (2020). Effect of shrinkage reducing admixture on drying shrinkage of concrete with different w/c ratios. Materials.

[30] Hamzah N., Mohd Saman H., Baghban M.H., Mohd Sam A.R., Faridmehr I., Muhd Sidek M.N., Benjeddou O., Huseien G.F. (2022). A review on the use of self-curing agents and its mechanism in high-performance cementitious materials. Buildings.

[31] Ramzi S., Hajiloo H. (2022). The effects of supplementary cementitious materials (SCMs) on the residual mechanical properties of concrete after exposure to high temperatures. Buildings.

[32] Park S., Wu S., Liu Z., Pyo S. (2021). The role of supplementary cementitious materials (SCMs) in ultra high performance concrete (UHPC): A review. Materials.

[33] Memon S.A., Javed U., Shah M.I., Hanif A. (2022). Use of processed sugarcane bagasse ash in concrete as partial replacement of cement: Mechanical and durability properties. Buildings.

[34] Fantilli A.P., Jóźwiak-Niedźwiedzka D. (2021). Supplementary cementitious materials in concrete, part I. Materials.

[35] Makul N. (2020). Effect of low-pressure microwave-accelerated curing on the drying shrinkage and water permeability of Portland cement pastes. Case Stud. Constr. Mater..

[36] Procházka M., Sobotka J., Šuhajda K., Novotný M. (2016). Microwave radiation and its application on construction materials. Eng. Struct. Technol..

[37] Novotny M., Skramlik J., Suhajda K., Tichomirov V. (2013). Sterilization of Biotic Pests by Microwave Radiation. Procedia Eng..

[38] Kvapilová V., Šuhajda K. (2020). Possibility of Using Microwave Radiation for Rehabilitation of Historical Masonry Constructions. Key Eng. Mater..

[39] Tauhiduzzaman M., Hafez I., Bousfield D., Tajvidi M. (2021). Modeling Microwave Heating and Drying of Lignocellulosic Foams through Coupled Electromagnetic and Heat Transfer Analysis. Processes.

[40] Průša D., Šuhajda K., Žajdlík T., Svobodová K., Šťastník S., Hobzova K., Venkrbec V. (2023). Effect of Microwave Radiation on the Compressive Strength of Solid Ceramic Brick. Buildings.

[41] Kääriäinen H., Rudolph M., Schaurich D., Tulla K., Wiggenhauser H. (2001). Moisture measurements in building materials with microwaves. NDT E Int..

[42] (2024). Plastics—Determination of Apparent Density of Material that can be Poured from a Specified Funnel.

[43] (2020). Thermal Insulating Products for Building Applications—Determination of Long-Term Water Absorption by Immersion.

[44] (1995). Determination of Thermal Conductivity of Building Materials and Products.

[45] Industry Statistics (2024). Cork Quality Council. https://www.corkqc.com/pages/industry-statistics.

[46] Yang C., Gao L., Liu X., Yang T., Yin G., Chen J., Guo H., Yu B., Cong H. (2019). Injectable Schiff base polysaccharide hydrogels for intraocular drug loading and release. J. Biomed. Mater. Res. A.

[47] Wu Z., Hong Y. (2019). Combination of the Silver-Ethylene Interaction and 3D Printing to Develop Antibacterial Superporous Hydrogels for Wound Management. ACS Appl. Mater. Interfaces.

[48] Farghaly Aly U., Aboutaleb H.A., Abdellatif A.A., Sameh Tolba N. (2019). Formulation and evaluation of simvastatin polymeric nanoparticles loaded in hydrogel for optimum wound healing purpose. Drug Des. Dev. Ther..

[49] Hu X., Tan H., Chen P., Wang X., Pang J. (2016). Polymer Micelles Laden Hydrogel Contact Lenses for Ophthalmic Drug Delivery. J. Nanosci. Nanotechnol..

[50] Pimenta A.F.R., Ascenso J., Fernandes J.C.S., Colaço R., Serro A.P., Saramago B. (2016). Controlled drug release from hydrogels for contact lenses: Drug partitioning and diffusion. Int. J. Pharm..

[51] De Sensale G.R., Goncalves A.F. (2014). Effects of Fine LWA and SAP as Internal Water Curing Agents. Int. J. Concr. Struct. Mater..

[52] Grzeszczyk S., Janus G. (2021). Lightweight Reactive Powder Concrete Containing Expanded Perlite. Materials.

[53] Leyton-Vergara M., Pérez-Fargallo A., Pulido-Arcas J., Cárdenas-Triviño G., Piggot-Navarrete J. (2019). Influence of Granulometry on Thermal and Mechanical Properties of Cement Mortars Containing Expanded Perlite as a Lightweight Aggregate. Materials.

[54] Szafraniec M., Barnat-Hunek D., Grzegorczyk-Frańczak M., Trochonowicz M. (2020). Surface Modification of Lightweight Mortars by Nanopolymers to Improve Their Water-Repellency and Durability. Materials.

[55] Ariyaratne I.E., Ariyanayagam A., Mahendran M. (2022). Bushfire-Resistant Lightweight Masonry Blocks with Expanded Perlite Aggregate. Fire.

[56] Beushausen H., Gillmer M., Alexander M. (2014). The influence of superabsorbent polymers on strength and durability properties of blended cement mortars. Cem. Concr. Compos..

